# Three-Dimensional Analysis of Vascular Development in the Mouse Embryo

**DOI:** 10.1371/journal.pone.0002853

**Published:** 2008-08-06

**Authors:** Johnathon R. Walls, Leigh Coultas, Janet Rossant, R. Mark Henkelman

**Affiliations:** 1 Mouse Imaging Centre (MICe), Hospital for Sick Children, Toronto Centre for Phenogenomics, Toronto, Ontario, Canada; 2 Department of Medical Biophysics, University of Toronto, Toronto, Ontario, Canada; 3 Hospital for Sick Children Research Institute, Developmental and Stem Cell Biology Program, Toronto, Ontario, Canada; 4 Department of Molecular Genetics, University of Toronto, Toronto, Ontario, Canada; University of Sydney, Australia

## Abstract

Key vasculogenic (*de-novo* vessel forming) and angiogenic (vessel remodelling) events occur in the mouse embryo between embryonic days (E) 8.0 and 10.0 of gestation, during which time the vasculature develops from a simple circulatory loop into a complex, fine structured, three-dimensional organ. Interpretation of vascular phenotypes exhibited by signalling pathway mutants has historically been hindered by an inability to comprehensively image the normal sequence of events that shape the basic architecture of the early mouse vascular system. We have employed Optical Projection Tomography (OPT) using frequency distance relationship (FDR)-based deconvolution to image embryos immunostained with the endothelial specific marker PECAM-1 to create a high resolution, three-dimensional atlas of mouse vascular development between E8.0 and E10.0 (5 to 30 somites). Analysis of the atlas has provided significant new information regarding normal development of intersomitic vessels, the perineural vascular plexus, the cephalic plexus and vessels connecting the embryonic and extraembryonic circulation. We describe examples of vascular remodelling that provide new insight into the mechanisms of sprouting angiogenesis, vascular guidance cues and artery/vein identity that directly relate to phenotypes observed in mouse mutants affecting vascular development between E8.0 and E10.0. This atlas is freely available at http://www.mouseimaging.ca/research/mouse_atlas.html and will serve as a platform to provide insight into normal and abnormal vascular development.

## Introduction

The cardiovascular system is the first functional organ system to develop in the mammalian embryo. The blood vessels that initially comprise this organ originate by vasculogenesis, the aggregation of de novo-forming angioblasts (endothelial precursors) into simple endothelial tubes. Angioblasts in the mouse embryo first emerge from the mesoderm as Flk1+ cells around embryonic day (E) 7.0 and assemble a simple circulatory loop consisting of a heart, dorsal aorta, yolk sac plexus and sinus venosus by E8.0 [1], [2], [3]. Shortly after its formation, this early vascular circuit is remodelled by angiogenesis, the proliferation, sprouting and pruning of pre-existing vessels, transforming it into a complex network of branched endothelial tubes of varying diameter, length and identity. Such remodelling of pre-existing vessels is dependent on both genetically hardwired events and hemodynamic forces [4], [5].

Given the complex nature of the vascular system and the diversity of biological processes required for its assembly and refinement, it is hardly surprising that a large number of signalling pathways are employed in its development. Mutations in pathways required for vascular development frequently manifest phenotypes that result in embryonic lethality at mid gestation. In mice, mutations affecting Notch [6], [7], [8], TGF*β* [9], [10], Hedgehog [11], [12], [13], VEGF [14], [15], [16], ephrin/Eph [17] and angiopoietin/Tie [18] signalling (among others) result in abnormal vascular development between E8.0 and E10.0 and ultimately embryonic lethality. The vascular activities of these pathways are not limited to this developmental time window, but extend to organogenesis [19], [20], maintenance of vascular homeostasis in adulthood [8], [9], [21], [22] and states of pathological angiogenesis [23], [24], [25], [26]. Correct interpretation of how these pathways regulate vascular development between E8.0 and E10.0 would therefore improve our understanding of how they contribute to later vascularization events. Such interpretation is often impeded however, by the complex nature of the vascular phenotypes, an inability to observe the vasculature of the mutants in its entirety and an incomplete understanding of the normal sequence of vascular remodelling events that occur during this period of development. Previous studies in zebrafish [27], [28] and chick [29], [30] have provided insight into normal vascular development, but have limited applicability to the sequence of vascular remodeling events in the mammalian embryo primarily due to differences in anatomy and the increased use of plexus bed intermediates in mammals compared to zebrafish. We have sought to address this issue by generating a high resolution, three-dimensional (3D) atlas of the developing mouse vasculature between E8.0 and E10.0 (5–30 somites).

The mouse embryo grows rapidly between E8.0 and E10.0 and undergoes complex morphological and conformational changes that present significant challenges to current imaging technologies. These challenges are further complicated by the inherent properties of the vascular system as a 3D network of branched, interconnected tubes of varying length and size. Accurate assessment of vascular development at this stage therefore requires a 3D imaging modality capable of visualizing the vasculature in its un-manipulated entirety in embryos of increasing size while retaining sufficient isotropic resolution (on the order of a few microns) to capture the details of the finest capillaries. Without these properties, significant positional information about the vasculature is lost and artefacts are introduced. While confocal microscopy has been used to generate an atlas of vascular development in zebrafish embryos [27] and study projections of the vasculature of dissected mouse embryos prior to E8.5 [2], it does not provide sufficient specimen coverage to create the 3D images necessary to visualize the complete embryonic vasculature in mouse embryos beyond this time point. Later embryonic stages have been studied using corrosion casting and electron microscopy [31]. While these investigations provided 3D representation, they did not retain information about vessels that cannot be perfused such as blind ended angiogenic sprouts, narrow vessels and vessels yet to form a complete lumen.

Recently, a new imaging modality named Optical Projection Tomography (OPT) [32] was developed to obtain molecular specificity and 3D cellular level resolution over complete specimens up to 1 cubic centimetre (cc) in size. OPT has been shown to support the use of multiple molecular markers [32], and has been previously used to visualize mouse embryos [32], [33], chick embryos [34], developing plant material [35], *drosophila melanogaster* [36] and whole adult mouse organs [37], [38]. We have further developed OPT using frequency-distance relationship [39] (FDR)-based deconvolution to obtain higher resolution images, on the order of a few microns, while still retaining the ability to image large specimen sizes [40], [41]. We set out to use this technique to study the normal sequence of mouse vascular development between E8.0 and E10.0.

Ideally one would be able to image a single living mouse embryo over an extended period of time in order to visualize the complete development of a single vasculature. 3D optical imaging of the live mouse embryo, however, is still a challenge due to the *in utero* development of mouse embryos and significant light scattering caused by living tissue. This *in vivo* time course can instead be approximated by static imaging of multiple genetically identical embryos collected at a range of ages throughout gestation. To this end, we performed FDR-based deconvolution OPT imaging on fixed PECAM-1-immunostained embryos ranging from 5 to 30 somites to study the normal development of the early embryonic mouse vasculature. PECAM-1 is major constituent of the endothelial cell intercellular junction and is widely used as a molecular marker for mature endothelial cells [2], [42], [43], [44]. With these images we are able to visualize vascular development across this age range, from vasculogenesis to capillary plexus to the development of larger vessels. The collection of all images comprises an atlas of normal development of the embryonic mouse vasculature, which is made freely available to other researchers. Analysis of this atlas has resulted in significant new information regarding the normal vascular development in the mouse embryo.

## Results and Discussion

Acronyms for the vessels discussed in this paper are listed in Table 1.

**Table 1:** Acronyms Used. The list of vessel acronyms used in this paper.

Acronym	Vessel
AVS	Arteriovenous shunt
ACV	Anterior cardinal vein
CCV	Common cardinal vein
DA	Dorsal aorta
DLAV	Dorsal longitudinal anastomotical vessel
ICA	Internal carotid artery
ISA	Intersomitic artery
ISV	Intersomitic vein
PCV	Posterior cardinal vein
PVH	Primary head vein
PMA	Primitive maxillary artery
PNVP	Perineural vascular plexus
OA	Omphalomesenteric artery
OV	Omphalomesenteric vein
UA	Umbilical artery
UV	Umbilical vein
SV	Sinus venosus
VTA	Vertebral artery

### 3D Visualization of the Embryonic Mouse Vasculature

**Figure 1:**
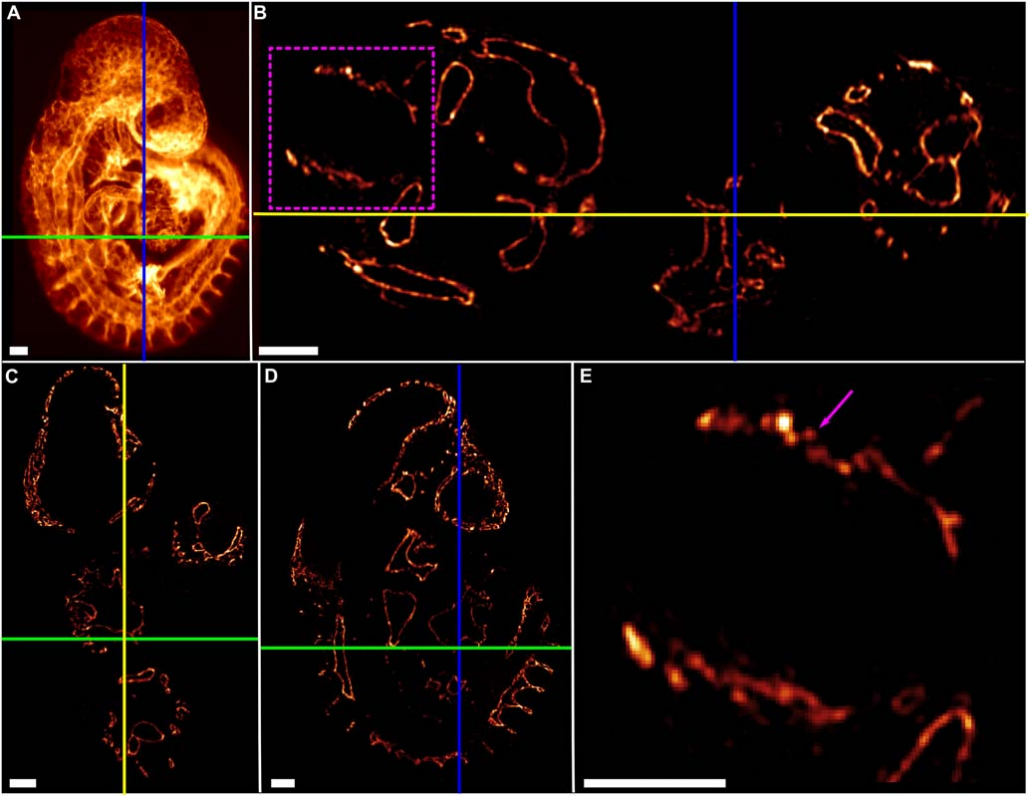
Example OPT view and slices from embryonic vascular mouse atlas data. (A) An example OPT view from a Cy3-PECAM-1 immunostained 19 somite mouse embryo demonstrating the complexity of the vascular pattern at this stage of development. The coloured lines (green, blue, yellow) in this and subsequent subfigures indicate the slice taken from the 3D reconstruction. (B,C,D) Slices from the reconstructed 3D FDR-deconvolution OPT data set can be viewed along any dimension. B is sliced along the green line in A, C is sliced along the blue line in A, and D is sliced along the yellow line in B and C. (E) Magnified view of the boxed region in B, depicting the perineural vascular plexus (pink arrow). All scale bars represent 100 microns.

A sample OPT view of the Cy3-PECAM-1 signal from a 19 somite embryo is shown in Figure 1. At 0.9 degree angular increments of the specimen, 400 views were taken throughout a complete revolution. These views were then FDR-filtered as described (see Materials and Methods) and reconstructed using a standard parallel-ray filtered backprojection reconstruction algorithm [45]. The resulting 3D data can be digitally sliced and viewed along any angle. Example slices from the three orthogonal axes indicated by the blue, yellow, and green lines in Figure 1A and B are shown in Figure 1B–D. The pixel size and slice thickness of the digital slices is 2.0 µm. The technique has sufficient resolution to visualize the finest vessel structures, such as those of the perineural vascular plexus, with an estimated diameter of 4 µm (Figure 1E).

**Figure 2:**
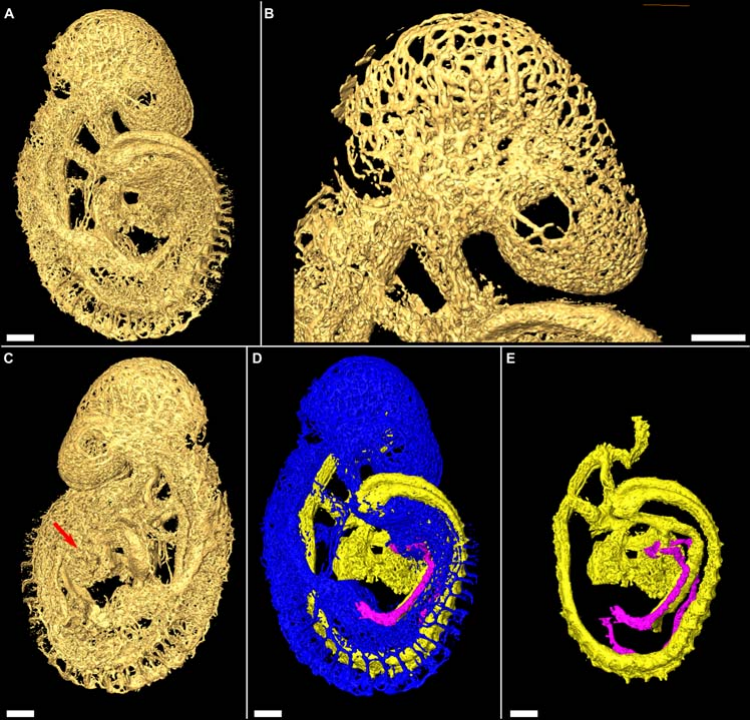
Surface renderings of embryonic vascular structures. (A) Reconstructed FDR-deconvolution OPT data of the 19 somite embryo is shown as a surface rendered object. (B) The surface rendered object can be zoomed in to any magnification, as in this magnified image of the vasculature in the mouse head. (C) The surface rendering can also be rotated so that it can be viewed from any angle. Viewing the rendering from the left side reveals structures in the heart (arrow) that are obscured by the tail in (A). (D) The 3D data can also be segmented as described in Materials and Methods. The DA, heart and ICAs are labelled yellow, the UV dark pink, and the unsegmented vasculature blue. (E) Segmentation of the data allows selective display of labelled structures. Exclusion of the unsegmented data provides better analysis of the ICAs and the pharyngeal arch arteries. All scale bars represent 100 microns.

Surfaces can be rendered from the 3D data set using an isosurface algorithm (see Materials and Methods) that creates a digital surface corresponding to constant intensity values in the reconstructed data. The resulting 3D surface contains those structures of the vascular network whose intensity values were greater than the chosen isovalue, which was selected to be approximately 1/4 the way between the background and maximum intensity value. The isosurface can be used to visualize the complete vasculature of the embryo as shown in Figure 2A. It can be zoomed in to give a magnified view of structures of interest, as in Figure 2B, and it can be arbitrarily rotated to view the surface from any angle, as illustrated in Figure 2C. The 3D data can also be manually segmented (see Materials and Methods), and rendered in different colours according to the segmentation label. This is demonstrated in Figure 2D which shows a surface rendering of the Cy3-PECAM-1 signal from a 19 somite embryo, with the dorsal aorta (DA), heart and internal carotid arteries (ICA) rendered in yellow, the umbilical vein (UV) in pink and the rest of the Cy3-PECAM-1 signal in blue. Any set of labels can be excluded from the rendering to simplify visualization of structures of interest. The removal of the vasculature in the head, for example, simplifies visualization of the ICA (Figure 2E). Much of the following data is represented in this way.

**Figure 3:**
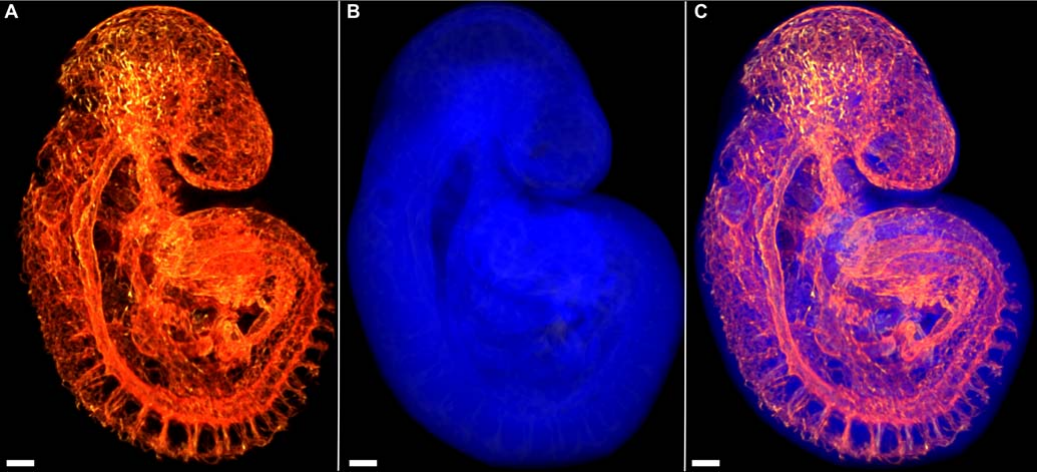
Volume renderings of embryonic vascular structures. (A) Reconstructed FDR-deconvolution OPT data can be visualized as a volume rendering. The vasculature of a 19 somite mouse embryo is visualized as a volume rendering using a hot metal colour map. (B) OPT data acquired for the mouse atlas include a co-registered 3D autofluorescence data set to visualize the vasculature in context of the rest of the embryo. The reconstruction from the OPT data of the autofluorescence channel of the 19 somite embryo can be visualized as a volume rendering using a blue colour map. (C) Since the data sets are co-registered, both volume renderings from (A) and (B) can be visualized in the same space. All scale bars represent 100 microns.

**Figure 4:**
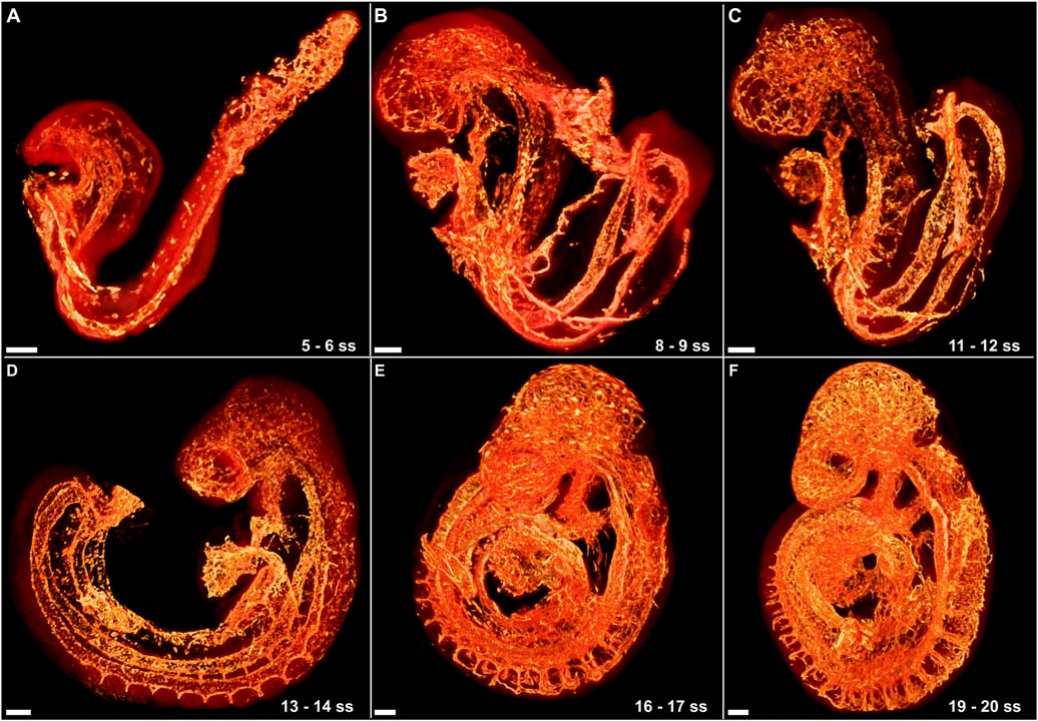
Stages of vascular development from 5 to 20 somites. (A) In the 5 somite embryo the vasculature, volume rendered with a hot metal colourmap, is confined mostly to a completed DA, a developing heart, the allantois, the extraembryonic circulation, and clusters of PECAM-1 expressing cells dispersed throughout the cephalic mesenchyme. The autofluorescence is volume rendered with a transparent red colour map for overall positioning. (B) The 8 somite embryo has a rudimentary vascular plexus permeating the cephalic mesenchyme, the UV is elongating and the heart has initiated looping. (C) Occipital intersomitic vessels have begun to develop in the 11 somite embryo. (D) After turning, the cervical intersomitic vessels emerge in the 14 somite embryo. (E) The intersomitic vessels have begun to branch and connect together in the 16 somite embryo. (F) The vasculature of the 19 somite mouse embryo is a complicated but stereotypic structure. All scale bars represent 100 microns.

Volumes can be alternatively rendered from the 3D data set using a direct volume rendering algorithm (see Materials and Methods) that assigns to each voxel arbitrarily selected emissive and absorptive properties according to its reconstructed intensity value and projects an image of the volume onto a 2D image plane. Volume renderings are particularly useful when visualizing co-registered 3D data sets, or when a 3D data set has small features or features with weak intensity values. A rendered volume can be used to visualize the Cy3-PECAM-1 signal and the nonspecific tissue autofluorescence separately (Figure 3A,B respectively) or, as the data sets are co-registered, simultaneously (Figure 3C). This process was performed on all 24 embryos ranging from 5 to 30 somites (Table 2), from which six are shown in Figure 4, to provide a complete map of PECAM-1 expression throughout the entire embryo. Reconstructions from embryos older than 20 somites had insufficient resolution to resolve the finest vascular details in the complete embryo, but were sufficient to position larger vessels and overall structure (data not shown).

**Table 2:** Embryos Imaged.

*Som #*	5–6	7–8	8–9	9–10	10–11	12–13	13–14	15–16	16–17	18–19	19–20	20+	Total
*n*	3	1	2	2	1	2	1	2	1	2	1	6	24

Twenty four (24) embryos were imaged in total across an age range from 5 to 30 somites.

These results demonstrate that the FDR-deconvolution OPT technique is capable of resolving the full range of vessels throughout whole embryos spanning the stages of 5–20 somites. Results presented below also illustrate one of the key benefits of the molecular specificity of FDR-deconvolution OPT as compared to imaging based on perfusion of the vascular lumen in that we were able to visualize endothelial cells and vasculature that is disconnected from the rest of the vascular tree and thus identify the origins of vessels at an earlier period of development. FDR-deconvolution OPT is the only imaging technique that has been shown to provide the combination of resolution, specimen coverage and molecular specificity necessary to image the complete vascular network in whole mouse embryos. Sufficient resolution is made possible in OPT imaging only by the inclusion of FDR-based deconvolution, which was shown to provide a factor of two resolution improvement over standard OPT imaging [41]. Our implementation of FDR-based deconvolution is performed entirely in software and can thus be used in conjunction with any OPT device without the need for hardware modification. FDR-deconvolution OPT is thus particularly well suited to the challenges presented by the developing vascular system of the mouse embryo and would be similarly suitable for imaging other detailed structures such as the developing nervous or lymphatic system.

We present here for the first time, a high-resolution three-dimensional atlas of the developing mouse vasculature in its native state between E8.0 and E10.0 of gestation (5–30 somites). The atlas comprises the collection of all 3D FDR-deconvolution OPT data sets of embryos ranging from 5 to 30 somites, as listed in Table 2. Videos of each of the data sets are made available at http://www.mouseimaging.ca/research/mouse_atlas.html. In the remainder of this paper, we present new information regarding the normal development of mouse vasculature that was obtained from analysis of the embryonic mouse vascular atlas.

### Vascular Development Between the 5 and 8 Somite Stage

**Figure 5:**
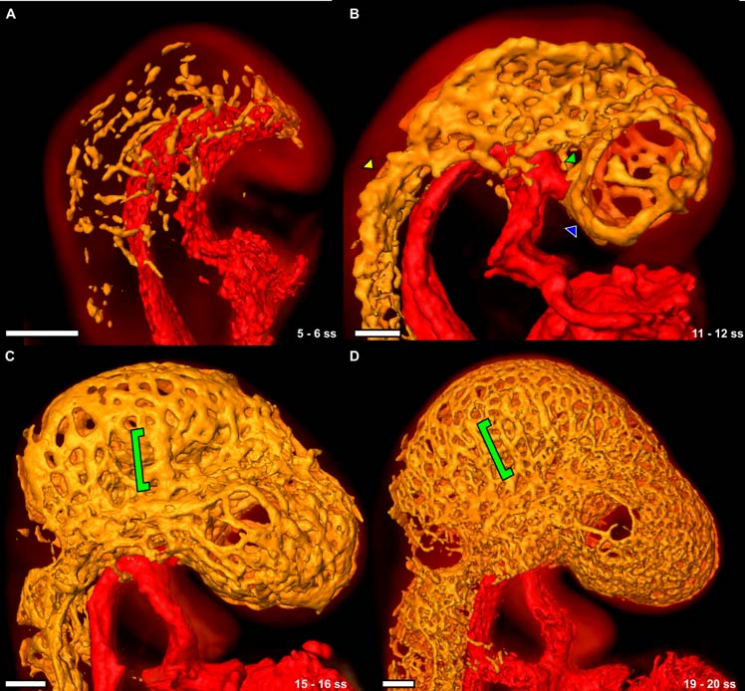
Development of the cephalic plexus between the 5 and 20 somite embryo. (A) The vasculature in the 5 somite mouse embryo is a series of disconnected clusters of PECAM-1-expressing cells. The DA and the heart are surface rendered red, PECAM-1 expression throughout the cephalic mesenchyme is surface rendered orange, and the autofluorescence of the mouse embryo is volume rendered with a hot metal colourmap. (B) By 11 somites, the cells have aggregated into a rudimentary vascular plexus. Larger vessels such as the PHV (blue arrowhead), the PMA (yellow arrowhead) and the ICA (green arrowhead) are visible (see also Supplemental Video S1). The PHV at this stage is a single large vessel that runs in an anterior-posterior direction starting from the cephalic flexure down to the first intersegmental vessel. (C) The cephalic plexus has remodelled into a more stereotypic pattern by 15 somites. The cephalic veins are easily distinguishable (green bracket). (D) At 19 somites the cephalic plexus has become more refined into recognizable structures. The cephalic veins are still visible at this stage (green bracket). All scale bars represent 100 microns.

Continuous PECAM-1 expression in the 5 somite embryo was confined to the completed dorsal aorta (DA), the heart and the allantois as previously reported [2]. Disconnected clusters of PECAM-1 expression were evident throughout the cephalic mesenchyme and lateral mesoderm at this age (Figure 5, Supplemental Video S1). These discrete clusters of PECAM-1 expressing cells were not connected to established vessels, suggesting they were locations of vasculogenesis rather than angiogenesis, consistent with observations that cephalic mesoderm has intrinsic angiogenic potential and contributes to the vasculature of the head [46].

By 7 somites, some PECAM-1 clusters in the cephalic mesoderm had begun to aggregate together forming a single larger vessel predominantly along the anterior-posterior axis and the future location of the primary head vein (PHV), while other cells remained as yet disconnected. By 11 somites virtually all PECAM-1 expression in the cephalic mesoderm was connected and formed a rudimentary vascular plexus that lined both the neural tube and the cephalic body wall. Recognizable structures such as the PHV, primitive maxillary artery (PMA) and the primitive internal carotid artery (ICA) were evident at this stage. Left-right communication between the two hemispheres of cephalic plexus was initiated at 7 somites by two vessels originating from the branch point of the PMA and the ICA, extending medially towards each other and forming a complete vessel by 13 somites (data not shown). By 14 somites the smaller vessels lateral to the plexus combined to form the anterior cardinal vein (ACV), as has been previously reported [44]. The plexus had also extended to surround the length of the neural tube and the otic vesicle, and the recognizable pattern of the mesencephalic artery and cephalic veins had begun to emerge in the cephalic body wall. This pattern continued to develop and refine up to the 20 somite stage.

### Connection of Embryonic and Extraembryonic Circulation

The extraembryonic circulation of the mouse embryo is divided into two components: the omphalomesenteric (vitelline) vessels and the umbilical vessels. The former connect the embryo proper with the yolk sac, while the latter connect the embryo proper with the feto-maternal interface in the placenta.

**Figure 6:**
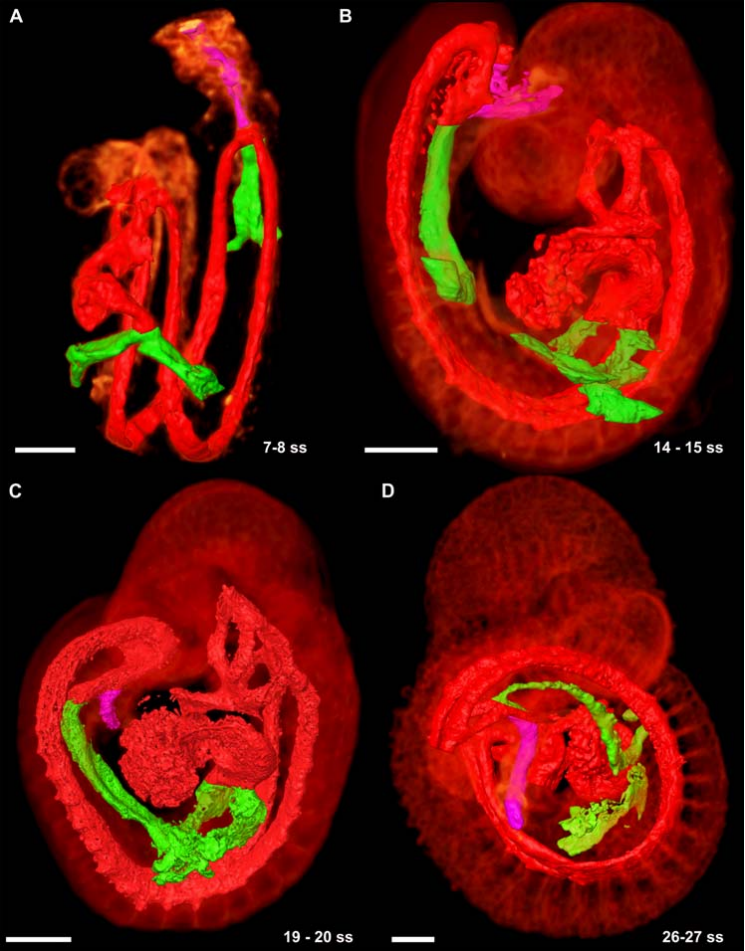
Connections between the embryonic and extraembryonic circulation in the early mouse embryo. (A) At 7 somites, the omphalomesenteric arteries and veins (green) are large structures. The UA (dark pink) connects to the DA and traverses the allantois, but is quite small. The DA and heart is surface rendered red, and the embryo autofluorescence volume rendered with a hot metal colourmap. The development of the UV is shown in Figure 6. (B) At 14 somites, the UA is accompanied by a series of smaller vessels connecting to nearby vasculature. At this point, it is still smaller than the OA and OV. After turning, the omphalomesenteric vessels branch off on one side of the embryo, and the umbilical vessels the other. (C) By 19 somites the UA has grown in size and is approximately equal in diameter to the OA. (D) By 26 somites, the OA is significantly smaller in diameter than the UA, suggesting that the balance of flow begins to favour the feto-maternal interface through the UA at some time between E9.0 and E9.5. All scale bars represent 250 microns.

The omphalomesenteric artery (OA) connects the yolk sac to the junction of the paired dorsal aorta at the most posterior tip of the embryo at E8.5. After turning, the OA leaves the embryo posterior to the developing heart, and eventually is folded back and fused to the dorsal aorta at E10.5 [47]. The OA has been previously reported to be present in the mouse as early as 7 somites [48]. We observed the OA as a single vessel in the 5 somite embryo that remained throughout the entire range of all embryonic stages studied and had folded back and fused to the DA by 30 somites (Figure 6).

The omphalomesenteric veins (OV) are paired vessels that connect the yolk sac to the sinus venosus (SV) of the embryo. The OV were located posterior to the developing heart throughout all stages imaged, initially draining directly into the SV (Figure 6A). The OV were exclusively extraembryonic until turning, at which point part of the OV traversed the future hepatic location in the embryo proper before connecting to the CCV. We observed the OV to be complete in the 5 somite embryo and present throughout the entire range of all embryonic stages studied (Figure 6).

The umbilical artery (UA) is initiated by de novo vasculogenesis in the allantois at E7.5 [49] and is of particular interest as it is implicated as a site of hematopoeitic stem cell development [50], [51], [52], [53], [54]. We observed the UA in the 5 somite embryo as a vessel fully formed throughout the allantois but unconnected to the dorsal aorta (data not shown). The UA in the allantois fused to the paired dorsal aorta at the base of the allantois by 7 somites (Figure 6A), consistent with previous reports of 6 somites [49], and remained an extraembryonic vessel through the entire range of all embryonic stages studied (Figure 6).

**Figure 7:**
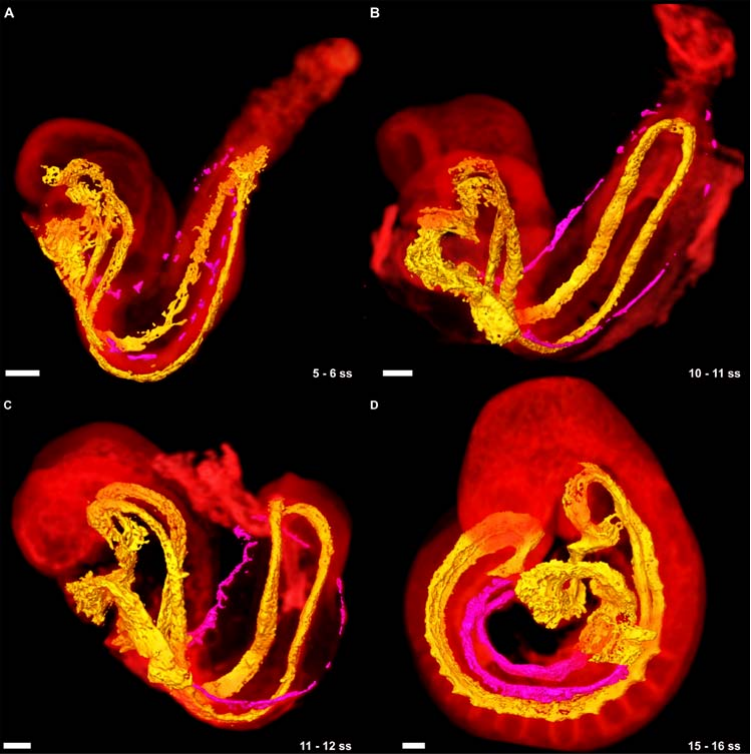
The development of the umbilical vein in the mouse embryo. (A) Discrete clusters of PECAM-1 expressing cells (dark pink) were evident along the length of the body wall immediately next to the junction of the body wall to amnion. The DA and heart is surface rendered yellow, and the embryo autofluorescence volume rendered with a hot metal colourmap. (B) The cells aggregated in a primarily anterior-posterior fashion beginning at the SV. (C) By 11 somites, the UV was almost complete, and had begun to develop a plexus which extended dorsolaterally. At the end of turning, the UV was complete and joined to the extraembryonic components of the vessel. (D) By 15 somites the UV is the second largest vessel in the embryo trunk. All scale bars represent 100 microns.

The umbilical vein (UV), unlike the UA, has embryonic as well as extraembryonic components, leading us to question its origins. We were able to trace the origins of the UV back to the 5 somite stage embryo. At this stage it was observed bilaterally as a disconnected string of PECAM-1 expressing cells at the junction of the body wall (ectoderm) and the amnion extending from the sinus venosus (SV) to the posterior tip of the embryo (Figure 7A). The disconnected nature of the Cy3-PECAM-1 signal indicated that the UV is formed by vasculogenesis. These cells then aggregated in a primarily anterior-posterior fashion to extend the length of the embryo (Figure 7B). As the embryo turned, the posterior end of the nearly completed UV was brought into contact with the base of the allantois, thereby allowing connection between the extraembryonic (allantoic) and embryonic portions (Figure 7C). By the end of turning (∼14 somites), the remaining cells had joined together to complete the UV as bilateral axial vessels running the length of the trunk from the SV through to the allantois (Figure 7D). At the 7 somite stage, several small branches were observed to extend from the rudimentary UV, which, by the 14 somite stage, developed into a capillary plexus permeating the body wall surrounding the intraembryonic coelom dorsal to the UV (data not shown). This plexus continued to develop along the UV with increasing age, eventually becoming continuous with either the rudimentary PCV or the intersomitic veins (see below). To our knowledge, this is the first report of how the embryonic portion of the UV is established. The rudiments of this vessel, along with the cephalic plexus described above, were likely present in the embryos examined by Drake and Fleming [2] in their study of vasculogenesis including embryos from 5 to 8 somites, however they would not likely have been recognized as such, in part due to partial dissection of the embryos to facilitate imaging and the limited nature of the 2D confocal imaging used. These findings demonstrate the value of our technique of maintaining the original morphology of the embryo and 3D imaging of developing structures in their entirety.

The 3D nature of FDR-deconvolution OPT data allows for measurements of vascular structures. We measured the relative diameter of the omphalomesenteric and umbilical vessels to serve as an indicator of relative blood flow volume. The diameter of the UA was less than that of the OA until the 19 somite stage embryo, at which point the two were approximately equal. By 28 somites, the diameter of the UA was approximately 1.5 times that of the OA. The UV, like the UA, were smaller in diameter than the OV until the 19 somite stage embryo, at which point the two were approximately equal. By the 26 somite stage, the UV was found to be approximately 1.5 times the diameter of the UV. Together, these results suggest that the volume of blood flow begins to favour the feto-maternal interface at approximately 20 somites or E9.0–E9.5, consistent with other data showing that the embryo becomes dependent on the chorioallantoic placenta by E10.0 [55].

### Intersomitic Vessels of the Occipital Region

Intersomitic (intersegmental) vessels are a useful model for sprouting angiogenesis and vessel pathfinding. They are the first vessels in the embryo to form by sprouting angiogenesis and their navigation between somites is guided by the same cues that guide axon growth cones (reviewed in [56], [57], [58], [59]). Intersomitic arteries and veins (ISA and ISV) are branches of the DA and PCV respectively that extend dorsally between the borders of their adjacent somites. The development of intersomitic vessels over time can be followed in a single embryo at a single time point as they emerge in a temporally regulated fashion along the anterior posterior axis from oldest to youngest, similar to their adjacent somites. Comparison of embryos at different stages of development revealed that there was a distinction in the development of intersomitic vessels bounded by the first 5 (occipital) somites compared to those bounded by trunk somites (at least for somites 6–20). As such we refer to these intersomitic vessels as “occipital” and “trunk” respectively.

**Figure 8:**
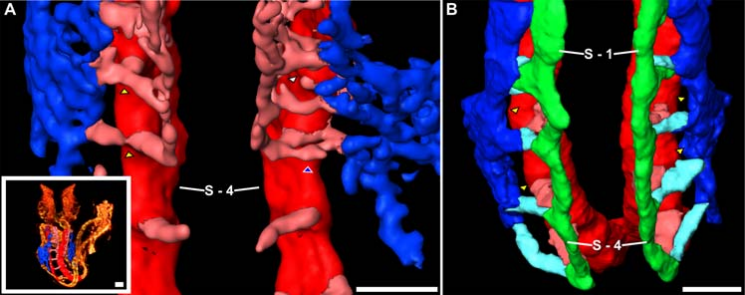
Development of the occipital intersomitic vessels. (A) The various stages of occipital intersomitic vessel development can be visualized in the 8 somite embryo (inset). The vessels surrounding somites 1 through 5 are segmented as DA (red), intersomitic vessels (pink) and the early cardinal vein (blue). The vessels initiate bilaterally, and an AVS originating from the DA connects to the cardinal vein (yellow arrowheads). An ISV then develops connecting the distal tip of the ISA to the cardinal vein (blue arrowhead). The ISA soon regresses (white arrowhead) leaving the vertebral artery connected to the cardinal vein. Somite number 4 is labelled as S-4. (B) The transient AVS have regressed (yellow arrowheads) by 15 somites, leaving only the expected dorsal ISA (pink), connected by the VTA (green), which connects via ISVs (light blue) to the ACV or CCV (blue). Somites numbers 1 and 4 are labelled as S-1 and S-4. All scale bars represent 100 microns.

We observed the occipital intersomitic vessels to consist of three interconnected vessels: a transient ISA, a transient arteriovenous shunt (AVS), and a persistent ISV. The ISAs formed first, initiating bilaterally as dorsal sprouts from the DA as early as 5 somites. At the level of each dorsal ISA, a lateral branch originating from the DA was observed to extend towards the CCV, eventually connecting the two major vessels (Figure 8A). This created a direct communication between the DA and CCV and comprised an AVS. Upon reaching the dorsal margin of the bounding somites, the distal tips of the ISAs branched longitudinally, fusing with the neighboring ISAs to form the vertebral artery (VTA). Shortly after VTA formation, the ISV emerged as a branch between the CCV and the ISA. We cannot comment on the origin of this vessel as we did not observe any instances when the ISV was connected to solely either the CCV or the ISA. The fusion of the ISV to the ISA created a temporary triangular vascular structure involving the ISA, the AVS and the ISV. This was a short-lived structure, as regression of the ISA and AVS quickly followed (Figure 8B). All ISAs and AVS of the occipital region had regressed by the 18 and 28 somite stage respectively, leaving the CCV connected to the VTA via an ISV and the DA fully separated from the VTA and CCV. Overall, development of the occipital intersomitic vessels was a rapid process as evidenced by the rarity of capture of intermediate developmental stages.

The existence of transient DA-CCV arteriovenous shunts in the development of occipital intersegmental vessels has not been previously reported and it is unclear why they form at all. AVS similar to the DA-CCV shunts have been noted in the course of normal embryonic mouse vascular development connecting the primordial ACV directly to the DA, anterior to the first somite [44]. We were able to confirm the existence of these AVS in the same location in embryos with 8 somites (data not shown). Like the transient AVS between the DA and CCV, the AVS between the DA and the ACV were no longer present beyond the 18 somite stage, and were presumably pruned from the vasculature.

These transient connections, especially those between the DA and CCV, may be causative of the arteriovenous malformations (AVMs) that arise in mice defective for Notch [6], [60], [61] and TGF*β* signalling [62], [63]. AVMs are miscommunications between arteries and veins that bypass the normal capillary plexus, resulting in the shunting of blood from the arterial circulation directly back to the venous circulation. Ink flow patterns and histological analysis suggested that AVMs in Notch and TGF*β* mutants involved a shunting of blood from the DA into the ACV and CCV [6], [60], [61], [62], [63]. Importantly, these AVMs were observed at an age by which transient connections between the DA and the ACV and CCV should have fully regressed, suggesting that the AVMs could have arisen from failed regression of these naturally occurring connections. This would imply that Notch and TGF*β* may be required for the coordinated regression of these naturally occurring connections between arterial and venous vessels. Notch and TGF*β* may accomplish this through their known role in regulating artery/vein identity [6], [60], [61], [62], [63]. Artery/vein identity has been proposed to prevent AVMs either by keeping arterial and venous progenitors separate during vasculogenesis [64] or by preventing “promiscuous fusions between naïve endothelial sprouts” from pre-existing arteries and veins [62]. If we are correct, then artery/vein identity may have a third role: promoting the regression of pre-existing connections between arterial and venous territory. As hemodynamic forces are known to influence vascular remodelling [4], [5] it would be necessary to test whether Notch and TGF*β* act downstream of blood flow to regulate such remodelling or are part of a true genetically pre-programmed event. Close examination of DA–CCV AVM shunt fate in Notch, TGF*β* and blood flow mutant embryos would help shed light on this.

### Intersomitic Arteries of the Trunk Region

**Figure 9:**
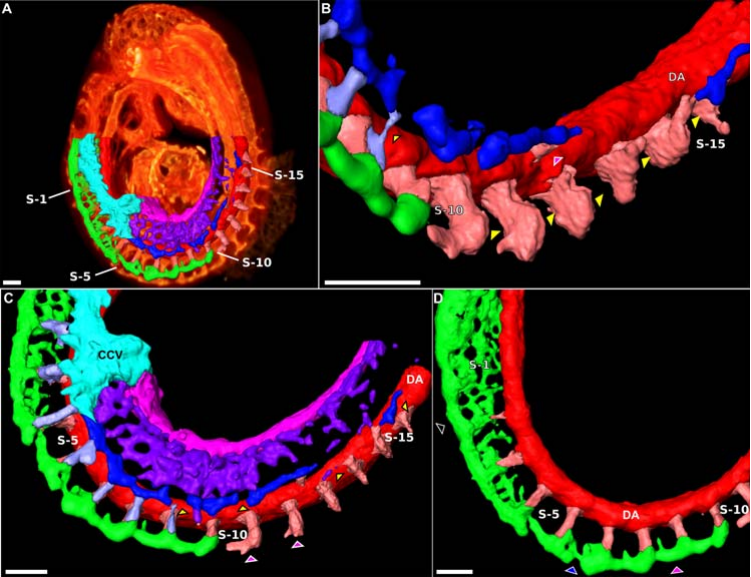
Development of the cervical intersomitic vessels. (A) The various stages of cervical intersomitic vessel development can be segmented as visualized as surface renderings in the 16 somite mouse embryo. The vessels along the right side of the embryo and surrounding somites 1 through 16 are labelled as: DA (red), ISA (pink), ISV (blue), VTA, DLAV and PNVP (green), ACV and CCV (cyan), UV (dark pink), UV plexus (purple), and PCV (blue). Somites 1, 5, 10 and 15 are numbered as S-1, S-5, S-10 and S-15. (B) Branches of PECAM-1 expression originating from the tips of the ISAs (yellow arrowheads) were observed to turn towards the location of the future PCV. (C) A second branch from the ISA was also observed to extend in a predominantly anterior direction (pink arrowheads) to connect up with other ISAs, eventually forming the DLAV. PECAM-1 expression along the location of the expected PCV was observed to lag development of the ISAs and is discontinuous (yellow arrowheads). (D) The PNVP develops through remodelling of the VTA and DLAV. Branches initiate medially from the DLAV (pink arrowhead), begin to remodel into simple mesh (blue arrowhead), and eventually remodel into a fine structured capillary plexus surrounding the neural tube. Note at this stage that the first ISA has regressed. Scale bars represent 100 microns.

Intersomitic vessels of the trunk differed from those of the occipital somites, both in their method of formation and final configuration. Trunk intersomitic vessels began to emerge bilaterally at the 8 somite stage as small dorsal protrusions in the paired DA, until the embryo began turning at the 11 somite stage (data not shown). Between this stage and the 12 somite stage (half turn), the protrusion at the level of the second trunk ISA (7th somite) had extended dorsally between the somites, and by 3/4 turning (13 somites), the protrusions down to the 11th somite had become extended branches. Subsequent ISA were observed to extend in more regulated intervals with advancing somite stage (Figure 9). Whereas zebrafish ISAs emerge from the aorta as narrow capillary-like projections composed of 3 or 4 linked endothelial cells [28], [65], each ISA in mouse appeared as a sheet-like evagination that was subsequently remodelled into a capillary-like ISA and DLAV (Figure 9). Unlike the occipital ISAs, transient branches directly connecting the DA and PCV were not observed, and ISAs were not seen to regress at any stage up to 30 somites. Distinct mechanisms of intersomitic vessel development in the occipital and trunk regions are fully consistent with differences in somitogenesis in these two regions. Unlike trunk somites, occipital somites do not form from presomitic mesoderm using the segmentation clock mechanism [66]. Furthermore, unlike trunk somites, occipital somites disperse and become undetectable as segments soon after their formation. As endothelial cells are highly responsive to their local microenvironment [67], differential patterning of occipital and trunk somites may explain why intersomitic vessels in the occipital region differ to those of the trunk.

### Intersomitic Veins and the Posterior Cardinal Vein

As the ISA approached the dorsal boundary of the somites, a branch was observed to extend laterally from the ISA toward the location of the future PCV, which is lateral to the DA (Figure 9B). This lateral branch became progressively more defined with age and formed the rudiment of the ISV, eventually connecting to either a rudimentary PCV or directly to the capillary plexus from the UV. A small number of branches of PECAM-1 expression were also observed to extend from the UV capillary plexus or the rudimentary PCV towards the rudimentary ISV. As for occipital ISA, each trunk ISA was paired with an accompanying ISV. This arrangement is in contrast to zebrafish, where the ISV sprouts from the PCV to join the ISA and in response to hemodynamic pressure, an alternating pattern of ISA/ISV is established [28].

PECAM-1 expression in the region of the expected PCV was first noted at 15 somites extending posteriorly from the CCV and connecting to the ISVs in a predominantly anterior-posterior fashion (Figure 9). Discontinuous PECAM-1 expression was observed along the path of the future PCV strongly suggesting its formation was a vasculogenic process, as occurs in the chick and zebrafish [29], [68]. Branches extended ventrally from the PCV and became continuous with the UV plexus as described above. By 16 somites PECAM-1 expression was continuous to the 8th ISV, extending discontinuously down to the 15th ISV, and by 19 somites the expression was continuous through all ISV. The vasculogenic activity in the vicinity of the PCV and its juxtaposition to the newly emerging ISVs makes it difficult to determine whether the ISV truly sprouts from its accompanying or is generated by (PECAM-1 negative) angioblasts recently added to the PCV by vasculogenesis. Detailed analysis of Flk1 expression as a marker of immature angioblasts combined with live imaging studies will be needed to resolve this issue.

PCV formation was disrupted in the vicinity of the developing limb bud (somites 8/9–13/14 [69]). At the 14 somite stage, an AVS connecting the DA to the rudimentary PCV was observed at the location of the 13th ISA, immediately posterior to the developing limb bud. This vessel was observed to develop bilaterally, but not necessarily at the same somite level. At the 20 somite stage, this vessel was still present in the same location, and at the 25 somite stage had developed into an artery feeding the developing limb. Additional branches were noted at this stage, originating from the DA and the neighbouring ISAs and connecting to the capillary plexus of the developing limb bud. The forelimb field, as defined by Tbx5 expression in lateral plate mesoderm, is first evident at the 8 somite stage [70]. We noted the limb bud itself to begin its formation at approximately 12 somites, and spanned the 8th to the 12th ISA. Together, these results suggest that the appearance of these limb arteries lags the development of the limb bud, and thus may be in response to a changing environment rather than a programmatic or preemptive occurrence.

### Anterior Branching of Intersomitic Arteries Establishes the DLAV

Upon reaching the dorsal margin of the somite and after formation of an ISV bud, trunk ISA tips branched longitudinally and fused with their adjacent ISAs forming the DLAV. This longitudinal branching was strongly biased in an anterior direction suggesting that it was guided by an attractive or repulsive mechanism (Figure 9C). The anterior bias of dorsal ISA branchings was not absolute, as some dorsal branches were seen to extend in a posterior direction, and in some cases bi-directionally. From 42 trunk ISAs for which branching directionality could be demonstrated, the number of anterior∶posterior∶bi-directional branching was 36∶4∶2, suggesting a strong bias for anterior branching of trunk ISAs. In the occipital region, ISAs observed were either completely connected to the VTA or had not yet begun to branch, thus we were unable to determine whether bias exists in occipital ISA branching. We observed instances of some trunk ISAs reaching the dorsal somite margin and initiating longitudinal branching prior to ISAs located anteriorly, indicating that timing of ISA remodelling is not absolute and is a highly dynamic process.

Anterior bias of trunk ISA branching is in contrast to the zebrafish, where ISAs branch in both anterior and posterior directions after reaching the dorsal boundary of the somite [28]. As described above, intersomitic vessel branching and pathfinding are regulated by the same guidance cues used by the axon growth cone. Somites are divided into rostral and caudal halves and many genes associated with axon guidance show polarized expression in these halves [71]. An anterior bias of ISA branching could be explained by attractive cues in the caudal half-somite anterior of the sprout or repulsive cues in the rostral half-somite posterior to the sprout.

EphrinB2 is one possible ISA attractant. In addition to being expressed in the ISA itself, ephrinB2 is expressed in the caudal half-somite during the time that longitudinal ISA branching is occurring [44], [72]. In vitro, ephrinB2 can induce endothelial sprouting [72], [73]. In vivo, ephrinB2−/− mice show defective vascular sprouting into the CNS [74] and reduced lymphatic sprouting [75], consistent with an attractive role for ephrinB2. While ephrinB2−/− mice display defective intersomitic vessel patterning that can be attributed to vascular specific ephrinB2 [44], these mutants were assessed after the DLAV had formed. Analysis of earlier time points in ephrinB2−/− embryos or ideally a somite specific ephrinB2 knockout would need to be performed to determine whether ephrinB2 is required for the anterior branching bias.

Our observations of preferential anterior branching toward the caudal half-somite would seem inconsistent with the expression of a known repulsive vascular cue, Sema3E, in this part of the somite [76]. One possibility is that Sema3E is not expressed early enough in the caudal half-somite to repel initial dorsal branching of the ISA, which we observed to occur before somites dispersed into sclerotome and dermamyotome. Sema3E expression in the caudal half-somite and defects in intersomitic vessel branching were reported at E10.5 and E11.5, after somites have begun to disperse and form sclerotome and dermamyotome compartments [76], [77]. Sema3E may therefore affect intersomitic branching that occurs subsequent to the primary branching we describe here. Similarly, the restriction of neural crest migration and peripheral nervous system axon pathfinding to the rostral half-somite occurs after formation of the sclerotome [71], [78], [79]. Guidance cues active in somites at this time such as the anti-angiogenic thrombospondin-1 in the rostral half-somite [80] are therefore not likely to be relevant to the vascular guidance we observed. Whatever the mechanisms are that govern the guidance of this branch, they are likely to involve a complex interplay of multiple attractive and repulsive factors, which together provide an anterior bias.

### Formation of the Perineural Vascular Plexus

The perineural vascular plexus (PNVP) is the precursor to the blood brain barrier and is recruited to surround the neural tube in response to VEGF between E8.5 and E9.5 [81], [82]. In mouse, vascular sprouts from the PNVP invade the neurepithelium around E10.0 in a stereotypic fashion [83], [84], [85]. PNVP sprouting into the neurepithelium is mediated by VEGF [86], ephrinB2 [74] and Tie1 [87], while subsequent branching and remodelling of the sprouts in the neuroepithelium is regulated by Np1 [85], heparin-binding VEGF isoforms [86], [88], Dll4/Notch [43] and netrin1/Unc5b [89] signalling. Failure of the PNVP to invade the neurepithelium results in neurodegeneration and neonatal lethality demonstrating the importance of this plexus to organogenesis [86].

The plexus was first evident around the 8 somite stage in the occipital somite region as rudimentary branches extending ventrally from the VTA, which by the 10 somite stage had extended as a capillary plexus around the neural tube at the level of the first somite. The PNVP in the cervical somite region (somites 6–12) was first observed at the 12 somite stage as rudimentary branches extending ventrally from the VTA, and then at the 16 somite stage as a capillary plexus extending from the VTA and DLAV and surrounding the neural tube at the level of the fourth somite (Figure 9D). The appearance of the rudimentary branches occurred soon after fusion of the VTA between two adjacent ISA. The PNVP extended to the 8th somite at the 20 somite stage, and down to the 20th somite by the 30 somite stage. Consistent with previous reports, the PNVP was first observed to invade the neural tube at the 27 somite stage (data not shown) [83], [84].

Quail-chick and mouse-quail chimera studies have shown that somites ([81] and references therein) and lateral mesoderm ([81] and references therein) are major sources of PNVP endothelial cells in the trunk. The fine chimerism between host and graft derived cells in the PNVP led the authors of one study to conclude that somite derived angioblasts migrated to and incorporated into the PNVP by a vasculogenic process [90]. While our study does not lend itself to fate mapping the cells comprising the PNVP, our results strongly suggest that during the stages we examined, the PNVP in the trunk remodels directly from the VTA and DLAV by angiogenesis, while in the cervical region it remodels from the cephalic plexus. As the VTA and DLAV originate from ISAs, which in turn arise from the dorsal wall of the DA, we would argue that the DA is the initial source of PNVP endothelial cells. In addition to the PNVP, somites [90], [91], [92], [93] and lateral mesoderm [92] both contribute to the dorsal wall of the DA and to ISAs in quail-chick chimeras. It is plausible to suggest that somite and lateral mesoderm contribution to the PNVP initially comes from a contribution to the DA, which subsequently donates its cells to the ISA and VTA/DLAV by angiogenesis. This would be consistent with zebrafish, where individual lateral mesoderm cells were found to migrate to the DLAV after incorporating into the DA and ISA [28], [65]. Somites may also make a second contribution to the PNVP, after the stages we imaged. Somite derived angioblasts may incorporate into or replace cells of the preformed PNVP as they have been demonstrated to do in the chick DA [92], [93], or, alternatively, somite derived vascular beds in the body wall may simply fuse to the pre-existing PNVP and contribute to it in that way.

### Conclusion

We have employed FDR-deconvolution OPT to generate a high-resolution three-dimensional atlas of the developing mouse vasculature in its native state between E8.0 and E10.0 of gestation (5–30 somites). Analysis of the 3D reference atlas we have constructed has revealed significant new information regarding normal development of the embryonic mouse vasculature. The need for an atlas such as this is critical, as numerous pathways required for vascular development exhibit severe vascular phenotypes during this time period when disrupted. This atlas can thus be used as a tool for better interpretation of these vascular phenotypes and as a platform to provide insight into normal mammalian vascular development. The observations in this paper represent only a portion of the information available in this atlas, which is provided for further study at http://www.mouseimaging.ca/research/mouse_atlas.html.

## Materials and Methods

### Embryo collection and Staining

Wild type ICR embryos were collected between the ages of embryonic day (E) 8.0 (5 somites) and E10.0 (30 somites). Noon of the plug day was considered to be E0.5. Embryos were dissected from their deciduas and Reichert's membranes, then, to maintain natural shape, were fixed for 1 h in 4% paraformaldehyde before remaining extraembryonic tissues were removed. For incompletely turned embryos, the amnion and the portion of yolk sac contiguous with the embryo were left attached to prevent disruption of embryonic-extraembryonic circulation. Embryos were then dehydrated through a graded series of methanol (25%, 50%, 75%, 100%) and stored at −20°C. Before staining, embryos were rehydrated and endogenous peroxidase activity was quenched with 3% H_2_O_2_. Non-specific antibody binding was blocked by pre-incubating embryos in 1% heat inactivated FCS (Hyclone, Logan UT) and 1% normal goat serum (Cedarlane, Burlington ON). Embryos were then stained overnight with 5 µg/mL anti-PECAM-1 antibody (Mec13.3) (BD Pharmingen). Primary antibody was detected by staining overnight with anti-rat HRP secondary antibody (Biosource, Camarillo CA) followed by incubation with tyramide-Cy3 reagent (1∶50) for 1 h (PerkinElmer, Boston MA). Experiments were approved by the Animal Care Committee of Mount Sinai Hospital (Toronto, ON, Canada) and were conducted in accordance with guidelines established by the Canadian Council on Animal Care.

Although all embryos presented in this study were processed according to the above protocol, we have since determined that methanol fixation slightly decreased the signal to noise ratio. While this effect did not significantly affect our imaging or findings, we would recommend replacing methanol fixation with a longer (4 h) paraformaldehyde fixation time followed by treatment with 50 mM sodium azide prior to quenching endogenous peroxidase by H_2_O_2_ treatment. A detailed description of the protocol is available at http://www.sickkids.ca/rossant/custom/protocols.asp.

### Optical Projection Tomography (OPT) of embryos

Optical projection tomography was performed as described previously [41]. Specimens were embedded in 1% low melting point (LMP) agarose and subsequently cleared using a 1∶2 mixture of benzyl alcohol and benzyl benzoate (BABB). The index-matched specimen was suspended from a stepper motor and immersed in a BABB bath with optically flat parallel glass windows. Images of the specimen were formed using a Leica MZFLIII stereozoom microscope equipped with a 0.5× objective lens and a 1.0× camera lens. Typical zoom settings used for image formation were between 4.0×–6.3×, resulting in numerical apertures from 0.0465 to 0.0620. Images (termed *views*) were acquired with a Retiga Exi CCD camera with pixel size 6.45×6.45 microns. Light from a mercury lamp was directed onto the specimen and filter sets were used to create fluorescent images of the specimen. An autofluorescence view was captured with the GFP1 filter set in the illumination and detection light path, and a view of the Cy3 fluorescence from the specimen was captured using the Cy3 filter set in the illumination and detection light path. The sample was rotated stepwise with a 0.9° step size through a complete revolution and views were acquired at each step.

Each OPT view approximates a parallel ray projection through the specimen. The temporal sequence from a row of detectors on the CCD forms a sinogram that is used to reconstruct the corresponding slice through the specimen using the standard convolution filtered back-projection algorithm [45]. The reconstruction of all slices yielded a 3D volumetric representation of the specimen. The stack of Cy3 views from a single specimen were subjected to Frequency Distance Relationship (FDR)-based filtering as described below, and both the filtered and unfiltered views reconstructed separately. The resulting 3D reconstruction of autofluorescence views and its corresponding 3D reconstruction of either filtered or unfiltered Cy3 views were co-registered.

### Point spread function acquisition

The point spread function of the optical system was required for the FDR-deconvolution process described below. A solution of silica beads (micromod sicastar-greenF 40-02-403) was mixed into 1% LMP agarose. A plug was cut out of the agarose and subjected to the same clearing process as the specimens. The plug was hung from the stepper motor, and images were acquired using the GFP1 filter set and the 4× zoom setting. The motorized focus moved the focal plane through the specimen, and an image of the bead plug was acquired at each step. An isolated bead was found in the stack. The data was resampled to approximate the PSF of the system at a wavelength of 600 nm rather than 535 nm, and at zoom settings of 5× and 6.3×.

### Frequency Distance Relationship (FDR)-based Deconvolution and Filtering

The stack of Cy3 views acquired over a complete revolution were subjected to Frequency Distance Relationship (FDR)-based deconvolution as described previously [41] and according to the equation
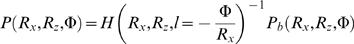
(1)where (*R_x_*, *R_z_*, Φ) is the Fourier equivalent of the sinogram space (*r_x_*, *r_z_*, *φ*). Specifically, (*r_x_*, *r_z_*) are the axes of detector element (perpendicular to the rotational axis) and detector row (parallel to the rotational axis) respectively, *l* is slope of the line in the (*R_x_*, Φ) plane and also the distance of the object from the lens, *P_b_*(*R_x_*, *R_z_*, Φ) is the 3D Fourier Transform (FT) of the blurred sinogram, and *P*(*R_x_*, *R_z_*, Φ) is the 3D FT of the unblurred sinogram. 
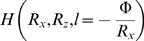
 is the FT of the distance dependent PSF, and is evaluated at each sample (*R_x_*, *R_z_*, Φ) using the FDR.

The filter *H* ^−1^ is constructed from four distinct components, as described in the equation
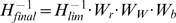
(2)where 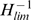 is a max-limited recovery filter designed according to the FDR using the experimentally acquired PSF, *W_r_* is a slope-based roll-off filter to exclude out of focus data, *W_W_* is a Wiener filter to deemphasize noise, and *W_b_* is a bandlimiting roll-off filter for high frequencies. The individual components are described in the equations:
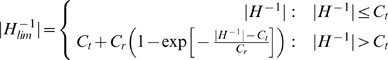
(3)

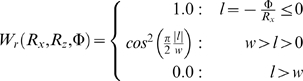
(4)

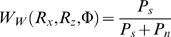
(5)and
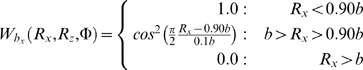
(6)with the parameters *C_t_* = 10, *C_r_* = 10, *w* = 0.3, and *b* set according to the NA of the system by the equation
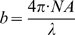
(7)assuming a wavelength λ = 630 nm.

Reconstructions resulting from the FDR based filtered projections were intensity normalized according to [41]. Both the filtered and unfiltered reconstructions were blurred by a 3D Gaussian with a full width at half-maximum of 40 pixels. The inverse of the ratio of the two blurred reconstructions was used as the ratio for intensity normalization of the filtered reconstruction.

The resolution achieved was estimated to be 5 microns at the 6.3× zoom setting, 6.5 microns at the 5× zoom setting, and 8 microns at the 4× zoom setting.

### Data visualization and segmentation

3D OPT reconstructions were loaded into Amira 3.1 (TGS, Inc.) for visualization. Surface renderings were created using the Amira “Isosurface” module with a threshold chosen just above the noise floor. Volume renderings were created using the Amira “Voltex” module, with the low threshold being chosen just above the noise floor and the high threshold chosen to maximize vessel visibility. Volume renderings of the autofluorescence reconstruction overlapping the Cy3-PECAM-1 reconstruction were created using a red colourmap for the autofluorescence and a hot metal colourmap for the Cy3-PECAM-1.

Reconstructions were segmented using the Amira module “LabelField” to maximize vessel visibility and aid in image interpretation. Observations were confirmed in both the unfiltered and filtered Cy3-PECAM-1 reconstructions. Surface renderings of the segmented vessels were performed using the same threshold but different colourmaps.

## Supporting Information

Video S1 Cephalic plexus of the 11 somite embryo. Larger vessels such as the PMA (blue) and the ICA (green) are easily distinguished in the cephalic plexus (orange). The DA and the heart are surface rendered red.
(10.68 MB MOV)

## References

[1] Ema M, Takahashi S, Rossant J (2006). Deletion of the selection cassette, but not cis-acting elements, in targeted Flk1-lacZ allele reveals Flk1 expression in multipotent mesodermal progenitors.. Blood.

[2] Drake CJ, Fleming PA (2000). Vasculogenesis in the day 6.5 to 9.5 mouse embryo.. Blood.

[3] Huber TL, Kouskoff V, Fehling HJ, Palis J, Keller G (2004). Haemangioblast commitment is initiated in the primitive streak of the mouse embryo.. Nature.

[4] le Noble F, Moyon D, Pardanaud L, Yuan L, Djonov V (2004). Flow regulates arterial-venous differentiation in the chick embryo yolk sac.. Development.

[5] Lucitti JL, Jones EAV, Huang CQ, Chen J, Fraser SE (2007). Vascular remodeling of the mouse yolk sac requires hemodynamic force.. Development.

[6] Gridley T (2007). Notch signaling in vascular development and physiology.. Development.

[7] Hofmann JJ, Iruela-Arispe ML (2007). Notch signaling in blood vessels - Who is talking to whom about what?. Circ Res.

[8] Niessen K, Karsan A (2007). Notch signaling in the developing cardiovascular system.. Am J Physiol Cell Physiol.

[9] Lebrin F, Deckers M, Bertolino P, Ten Dijke P (2005). Tgf-*β* receptor function in the endothelium.. Cardiovasc Res.

[10] Rossant J, Howard L (2002). Signaling pathways in vascular development.. Annu Rev Cell Dev Bio.

[11] Astorga J, Carlsson P (2007). Hedgehog induction of murine vasculogenesis is mediated by Foxf1 and Bmp4.. Development.

[12] Byrd N, Becker S, Maye P, Narasimhaiah R, St-Jacques B (2002). Hedgehog is required for murine yolk sac angiogenesis.. Development.

[13] Vokes SA, Yatskievych TA, Heimark RL, McMahon J, McMahon AP (2004). Hedgehog signaling is essential for endothelial tube formation during vasculogenesis.. Development.

[14] Coultas L, Chawengsaksophak K, Rossant J (2005). Endothelial cells and VEGF in vascular development.. Nature.

[15] Carmeliet P, Ferreira V, Breier G, Pollefeyt S, Kieckens L (1996). Abnormal blood vessel development and lethality in embryos lacking a single VEGF allele.. Nature.

[16] Ferrara N, Carver-Moore K, Chen H, Dowd M, Lu L (1996). Heterozygous embryonic lethality induced by targeted inactivation of the VEGF gene.. Nature.

[17] Kuijper S, Turner CJ, Adams RH (2007). Regulation of angiogenesis by Eph-ephrin interactions.. Trends Cardiovasc Med.

[18] Thurston G (2003). Role of angiopoietins and tie receptor tyrosine kinases in angiogenesis and lymphangiogenesis.. Cell Tissue Res.

[19] Pola R, Ling LE, Silver M, Corbley MJ, Kearney M (2001). The morphogen Sonic hedgehog is an indirect angiogenic agent upregulating two families of angiogenic growth factors.. Nat Med.

[20] White AC, Lavine KJ, Ornitz DM (2007). FGF9 and SHH regulate mesenchymal Vegfa expression and development of the pulmonary capillary network.. Development.

[21] Lee S, Chen TT, Barber CL, Jordan MC, Murdock J (2007). Autocrine VEGF signaling is required for vascular homeostasis.. Cell.

[22] Eremina V, Baelde HJ, Quaggin SE (2007). Role of the VEGF—a signaling pathway in the glomerulus: evidence for crosstalk between components of the glomerular filtration barrier.. Nephron Physiol.

[23] Ferrara N, Kerbel RS (2005). Angiogenesis as a therapeutic target.. Nature.

[24] Thurston G, Noguera-Troise I, Yancopoulos G (2007). The Delta paradox: DLL4 blockade leads to more tumour vessels but less tumour growth.. Nat Rev Cancer.

[25] Hellström M, Phng LK, Hofmann J, Wallgard E, Coultas L (2007). Dll4 signalling through Notch1 regulates formation of tip cells during angiogenesis.. Nature.

[26] Lobov IB, Renard RA, Papadopoulos N, Gale NW, Thurston G (2007). Delta-like ligand 4 (Dll4) is induced by VEGF as a negative regulator of angiogenic sprouting.. Proc Natl Acad Sci U S A.

[27] Isogai S, Horiguchi M, Weinstein BM (2001). The vascular anatomy of the developing zebrafish: an atlas of embryonic and early larval development.. Dev Biol.

[28] Isogai S, Lawson ND, Torrealday S, Horiguchi M, Weinstein BM (2003). Angiogenic network formation in the developing vertebrate trunk.. Development.

[29] Coffin JD, Poole TJ (1988). Embryonic vascular development: immunohistochemical identification of the origin and subsequent morphogenesis of the major vessel primordia in quail embryos.. Development.

[30] Poole TJ, Coffin JD (2005). Vasculogenesis and angiogenesis: Two distinct morphogenetic mechanisms establish embryonic vascular pattern.. J Exp Zool.

[31] Hiruma T, Nakajima Y, Nakamura H (2002). Development of pharyngeal arch arteries in early mouse embryo.. J Anat.

[32] Sharpe J, Ahlgren U, Perry P, Hill B, Ross A (2002). Optical projection tomography as a tool for 3D microscopy and gene expression studies.. Science.

[33] Lickert H, Takeuchi JK, Von Both I, Walls JR, McAuliffe F (2004). Baf60c is essential for function of BAF chromatin remodelling complexes in heart development.. Nature.

[34] Fisher ME, Clelland AK, Bain A, Baldock RA, Murphy P (2008). Integrating technologies for comparing 3D gene expression domains in the developing chick limb.. Dev Biol.

[35] Lee K, Avondo J, Morrison H, Blot L, Stark M (2006). Visualizing plant development and gene expression in three dimensions using optical projection tomography.. Plant Cell.

[36] McGurk L, Morrison H, Keegan LP, Sharpe J, O'Connel MA (2007). Three-dimensional imaging of Drosophila melanogaster.. PLoS ONE.

[37] Alanentalo T, Asayesh A, Morrison H, Lorén CE, Holmberg D (2007). Tomographic molecular imaging and 3D quantification within adult mouse organs.. Nat Methods.

[38] Hajihosseini MK, De Langhe S, Lana-Elola E, Morrison H, Sparshott N (2008). Localization and fate of Fgf10-expressing cells in the adult mouse brain implicate Fgf10 in control of neurogenesis.. Mol Cell Neurosci.

[39] Xia W, Lewitt RM, Edholm PR (1995). Fourier correction for spatially variant collimator blurring in SPECT.. IEEE Trans Med Im.

[40] Walls JR, Sled JG, Sharpe J, Henkelman RM (2005). Correction of artefacts in optical projection tomography.. Phys Med Biol.

[41] Walls JR, Sled JG, Sharpe J, Henkelman RM (2007). Resolution improvement in optical projection tomography.. Phys Med Biol.

[42] Chaturvedi K, Sarkar DK (2006). Isolation and characterization of rat pituitary endothelial cells.. Neuroendocrinology.

[43] Suchting S, Freitas C, le Noble F, Benedito R, Bréant C (2007). The Notch ligand Delta-like 4 negatively regulates endothelial tip cell formation and vessel branching.. Proc Natl Acad Sci U S A.

[44] Gerety SS, Anderson DJ (2002). Cardiovascular ephrinb2 function is essential for embryonic angiogenesis.. Development.

[45] Slaney M, Kak AC (1988). Principles of Computerized Tomographic Imaging.

[46] Couly G, Coltey P, Eichmann A, Ledouarin NM (1995). The angiogenic potentials of the cephalic mesoderm and the origin of brain and head blood-vessels.. Mech Dev.

[47] Garciaporrero JA, Godin IE, Dieterlen-Lièvre F (1995). Potential intraembryonic hemogenic sites at pre-liver states in the mouse anatomy and embryology.. Anat and Embryol.

[48] Wood HB, May G, Healy L, Enver T, Morriss-Kay GM (1997). CD34 expression patterns during early mouse development are related to modes of blood vessel formation and reveal additional sites of hematopoiesis.. Blood.

[49] Inman KE, Downs KM (2007). The murine allantois: emerging paradigms in development of the mammalian umbilical cord and its relation to the fetus.. Genesis.

[50] de Bruijn MF, Ma X, Robin C, Ottersbach K, Sanchez MJ (2002). Hematopoietic stem cells localize to the endothelial cell layer in the midgestation mouse aorta.. Immunity.

[51] Li Z, Chen MJ, Stacy T, Speck NA (2006). Runx1 function in hematopoiesis is required in cells that express Tek.. Blood.

[52] de Bruijn MFTR, Speck NA, Peeters MCE, Dzierzak E (2000). Definitive hematopoietic stem cells first develop within the major arterial regions of the mouse embryo.. EMBO Journal.

[53] Gekas C, Dieterlen-Lièvre F, Orkin SH, Mikkola H (2005). The placenta is a niche for hematopoietic stem cells.. Dev Cell.

[54] Ottersbach K, Dzierzak E (2005). The murine placenta contains hematopoietic stem cells within the vascular labyrinth region.. Dev Cell.

[55] Mu J, Adamson SL (2006). Developmental changes in hemodynamics of uterine artery, utero- and umbilicoplacental, and vitelline circulations in mouse throughout gestation.. Am J Physiol Heart Circ Physiol.

[56] Eichmann A, Makinen T, Alitalo K (2005). Neural guidance molecules regulate vascular remodeling and vessel navigation.. Genes Dev.

[57] Carmeliet P, Tessier-Lavigne M (2005). Common mechanisms of nerve and blood vessel wiring.. Nature.

[58] Suchting S, Bicknell R, Eichmann A (2006). Neuronal clues to vascular guidance.. Exp Cell Res.

[59] Jones C, Li DY (2007). Common cues regulate neural and vascular patterning.. Curr Opin Genet Dev.

[60] Krebs LT, Shutter JR, Tanigaki K, Honjo T, Stark KL (2004). Haploinsufficient lethality and formation of arteriovenous malformations in notch pathway mutants.. Genes Dev.

[61] Duarte A, Hirashima M, Benedito R, Trindade A, Diniz P (2004). Dosage-sensitive requirement for mouse dll4 in artery development.. Genes Dev.

[62] Sorensen LK, Brooke BS, Li DY, Urness LD (2003). Loss of distinct arterial and venous boundaries in mice lacking endoglin and a vascular-specific TGF-*β* coreceptor.. Dev Biol.

[63] Urness LD, Sorensen LK, Li DY (2000). Arteriovenous malformations in mice lacking activin receptor-like kinase-1.. Nat Genet.

[64] Lawson ND, Weinstein BM (2002). Arteries and veins: making a difference with zebrafish.. Nat Rev Genet.

[65] Childs S, Chen JN, Garrity DM, Fishman MC (2002). Patterning of angiogenesis in the zebrafish embryo.. Development.

[66] Dale K, Pourquié O (2000). A clock-work somite.. Bioessays.

[67] Nikolova G, Lammert E (2003). Interdependent development of blood vessels and organs.. Cell Tissue Res.

[68] Zhong TP, Childs S, Leu JP, Fishman MC (2001). Gridlock signalling pathway fashions the first embryonic artery.. Nature.

[69] Burke AC, Nelson CE, Morgan BA, Tabin C (1995). Hox genes and the evolution of vertebrate axial morphology.. Development.

[70] Agarwal P, Wylie JN, Galceran J, Arkhitko O, Li C (2003). Tbx5 is essential for forelimb bud initiation following patterning of the limb field in the mouse embryo.. Development.

[71] Kuan CY, Tannahill D, Cook GM, Keynes RJ (2004). Somite polarity and segmental patterning of the peripheral nervous system.. Mech Dev.

[72] Adams RH, Wilkinson GA, Weiss C, Diella F, Gale N (1999). Roles of ephrinB ligands and EphB receptors in cardiovascular development: demarcation of arterial/venous domains, vascular morphogenesis, and sprouting angiogenesis.. Genes Dev.

[73] Zhang XQ, Takakura N, Oike Y, Inada T, Gale N (2001). Stromal cells expressing ephrin-b2 promote the growth and sprouting of ephrin-b2(+) endothelial cells.. Blood.

[74] Wang HU, Chen ZF, Anderson DJ (1998). Molecular distinction and angiogenic interaction between embryonic arteries and veins revealed by ephrin-b2 and its receptor eph-b4.. Cell.

[75] Makinen T, Adams RH, Bailey J, Lu Q, Ziemiecki A (2005). Pdz interaction site in ephrinb2 is required for the remodeling of lymphatic vasculature.. Genes Dev.

[76] Gu C, Yoshida Y, Livet J, Reimert DV, Mann F (2005). Semaphorin 3e and plexin-d1 control vascular pattern independently of neuropilins.. Science.

[77] Kaufman MH, Bard JBL (1999). The Anatomical Basis of Mouse Development.

[78] Krull CE (2001). Segmental organization of neural crest migration.. Mech Dev.

[79] Sauka-Spengler T, Bronner-Fraser M (2006). Development and evolution of the migratory neural crest: a gene regulatory perspective.. Curr Opin Genet Dev.

[80] Tucker RP, Hagios C, Chiquet-Ehrismann R, Lawler J, Hall RJ (1999). Thrombospondin-1 and neural crest cell migration.. Dev Dyn.

[81] Hogan KA, Ambler CA, Chapman DL, Bautch VL (2004). The neural tube patterns vessels developmentally using the vegf signaling pathway.. Development.

[82] Evans HM (1909). On the development of the aortae and cardinal and umbilical veins and and the other blood vessels of vertebrate embryos from capillaries.. Anat Rec.

[83] Nagase T, Nagase M, Yoshimura K, Fujita T, Koshima I (2005). Angiogenesis within the developing mouse neural tube is dependent on sonic hedgehog signaling: possible roles of motor neurons.. Genes Cells.

[84] Nakao T, Ishizawa A, Ogawa R (1988). Observations of vascularization in the spinal-cord of mouse embryos, with special reference to development of boundary membranes and perivascular spaces.. Anat Rec.

[85] Gerhardt H, Ruhrberg C, Abramsson A, Fujisawa H, Shima D (2004). Neuropilin-1 is required for endothelial tip cell guidance in the developing central nervous system.. Dev Dyn.

[86] Haigh JJ, Morelli PI, Gerhardt H, Haigh K, Tsien J (2003). Cortical and retinal defects caused by dosage-dependent reductions in vegf-a paracrine signaling.. Dev Biol.

[87] Sato TN, Tozawa Y, Deutsch U, Wolburg-Buchholz K, Fujiwara Y (1995). Distinct roles of the receptor tyrosine kinases tie-1 and tie-2 in blood vessel formation.. Nature.

[88] Ruhrberg C, Gerhardt H, Golding M, Watson R, Ioannidou S (2002). Spatially restricted patterning cues provided by heparin-binding VEGF-A control blood vessel branching morphogenesis.. Genes Dev.

[89] Lu X, Le Noble F, Yuan L, Jiang Q, De Lafarge B (2004). The netrin receptor unc5b mediates guidance events controlling morphogenesis of the vascular system.. Nature.

[90] Ambler CA, Nowicki JL, Burke AC, Bautch VL (2001). Assembly of trunk and limb blood vessels involves extensive migration and vasculogenesis of somite-derived angioblasts.. Dev Biol.

[91] Wilting J, Brand-Saberi B, Huang R, Zhi Q, Kontges G (1995). Angiogenic potential of the avian somite.. Dev Dyn.

[92] Pardanaud L, Luton D, Prigent M, Bourcheix L, Catala M (1996). Two distinct endothelial lineages in ontogeny, one of them related to hemopoiesis.. Development.

[93] Pouget C, Gautier R, Teillet MA, Jaffredo T (2006). Somite-derived cells replace ventral aortic hemangioblasts and provide aortic smooth muscle cells of the trunk.. Development.

